# Changes in mutation frequency of eight Mendelian inherited disorders in eight pedigree dog populations following introduction of a commercial DNA test

**DOI:** 10.1371/journal.pone.0209864

**Published:** 2019-01-16

**Authors:** T. W. Lewis, C. S. Mellersh

**Affiliations:** 1 The Kennel Club, London, England; 2 School of Veterinary Medicine and Science, The University of Nottingham, Sutton Bonington Campus, Sutton Bonington, Leicestershire, England; 3 The Kennel Club Genetics Centre at the Animal Health Trust, Kentford, Newmarket, Suffolk, England; University of Illinois, UNITED STATES

## Abstract

**Introduction:**

DNA testing for autosomal recessive disease mutations in many dog breeds is now relatively commonplace. There have, however, been few efforts made to determine changes in the frequency of disease causing mutations as a result of probable selection based on the results of DNA testing. This study makes use of genotype data from both DNA test results reported to the UK Kennel Club and where known from a ‘hereditary status’ (where a definitive genotype may be inferred and ascribed based on known parental genotypes) to do so.

**Results:**

The results, using all known genotype data, show a general and sizeable decline in disease causing mutation frequency across eight diseases in eight breeds (by between 12–86% in dogs born 2–4 years after publication of the mutation, and by nearly 90% or more in those born 8–10 years after). In contrast, data from test results only, while revealing an almost complete and immediate end to the production of affected individuals, show little general decline in either the derived mutation frequency or the proportion of heterozygote carriers. It appears that the numerical size of the breed is an important determinant on the rate of uptake of a DNA test (as judged by the proportion of a breed born four years after publication of the disease-causing mutation with a known genotype).

**Conclusion:**

These results show that dog breeders appear to be incorporating the results of DNA testing into their selection strategies to successfully decrease the frequency of the mutation. It is shown that use of DNA test result data alone does not reveal such trends, possibly as some breeders undertake testing to determine clear stock which can then be used to produce future disease-free generations in the knowledge they are not carrying the disease causing mutation.

## Introduction

More than 700 inherited disorders and traits have been described in the domestic dog [1]. Approximately 300 of these are estimated to have a genetically simple (Mendelian) mode of inheritance with the causal mutations for over 240 Mendelian disorders and traits (such as coat colour) having been identified to date [1]. The precise number of globally available canine DNA tests that are based on specific disease-associated mutations is unknown but is likely to be well in excess of 150 [2]. The evolutionary and recent history, population structure and selection practices of domestic dog populations are posited as the cause of the large number of autosomal recessive mutations at relatively high frequencies across various dog breeds [2, 3]. The genotypes provided by DNA testing improve the accuracy of selection against a fully-penetrant autosomal recessively inherited disease by enabling breeders to definitively identify among potential breeding candidates: i) homozygotes for a non-disease causing (wild type) allele (often referred to as *unaffected* or *clear*) ii) heterozygotes (typically referred to as *carriers)* and iii) homozygotes for the disease-causing mutation (typically referred to as *affected*) prior to the development of clinical signs of disease. Since the rate of *de novo* mutation occurs at a negligible rate (the average mammalian genome mutation rate reported as 2.2x10^-9^ per base pair per year; [4]) the risk of a repeat mutation occurring (the exact same mutation occurring more than once in the exact same position) can safely be ignored, and the genotypes of progeny of parental genotype combinations are predictable. Breeders may then use this information to ensure no puppies homozygous for the disease causing mutation, (and thus destined to be affected by the disease) are born.

In some cases, therefore, a dog’s genotype may definitively be deduced from the genotypes of its parents, when they are known. For example, in the case of an autosomal recessive disorder, all progeny of tested ‘clear’ and ‘affected’ parents will be heterozygous for the wild type and mutation, and so will be ‘carriers’. Importantly, progeny of two ‘clear’ parents will also be ‘clear’. The Kennel Club, which records canine parentage or pedigree information together with results of health screening and DNA testing of UK dogs contained in the pedigree, has facilitated assignment of ‘hereditary’ status to such dogs of determinable genotype, which are 1) progeny of two clear parents, 2) progeny of two affected parents, and 3) progeny of one clear and one affected parent. This has resulted in the propagation of ‘hereditary clear’ status across generations for dogs whose breeders have invested in genetic testing and avoids the costs associated with testing every successive generation to confirm a known genotype.

Previous studies investigating DNA testing patterns have examined results of the DNA tested population, determining genotype and allele frequencies, but have been unable to take account of the increasing cohort of the population established to be free from the mutation; the untested but ‘hereditary clear’ puppies born over successive generations [2, 5, 6]. As a result, such data are not reflective of the population and will skew estimates of mutation frequency and hamper the ability to detect any change as a consequence of selection. In this paper, we make use of the ‘hereditary’ status of Kennel Club registered dogs, alongside results from the DNA tested population, to derive frequency estimates for mutations causing a range of diseases in a number of breeds, which is more representative of the total breeding population.

## Methods

The results of DNA tests as reported to the Kennel Club, for all individual dogs born up to and including 31^st^ December 2017 for the breeds and disorders listed in Table 1 were used in this study. The breeds and disorders were chosen to reflect variation in numerical breed size and frequency of the disease-causing mutation at the time of publication. The data included the results of dogs that had undergone a DNA test themselves and also those assigned a hereditary status based on the DNA test results of their ancestors. Results of DNA tests are routinely provided directly to the Kennel Club by many commercial test providers (CTPs) to enable public listing in the Kennel Club’s ‘Health Test Results Finder’ web-tool (https://www.thekennelclub.org.uk/services/public/mateselect/test/Default.aspx), which displays the results of DNA tests and health tests pertinent to the breed for an individual named dog (the Kennel Club name ‘Elkayess Grace Jeddler’ may be entered to view the reporting of results). In some cases, where CTPs do not directly forward results to the Kennel Club, owners may submit the results themselves. Publication of DNA testing and health screening results on the ‘Health Test Results Finder’ allows verification of testing and health screening results independently of the owner/breeder. Many owners/breeders participating in health testing are keen for their results to be publically available in this manner.

**Table 1:** Study breeds and disorders.

DNA test	Gene	OMIA ref	Mutation published [ref]	Breed (abbreviation)
Progressive rod-cone degeneration (prcd-PRA)	PRCD	001298–9615	August 2006 [7]	Labrador Retriever (LAB)
				Cocker Spaniel (CKR)
Early onset [hereditary] cataract (HC)	HSF4	001758-9615	September 2006 [8]	Staffordshire Bull Terrier (SBT)
Exercise induced collapse (EIC)	DNM1	001466-9615	October 2008 [9]	Labrador Retriever (LAB)
Lens luxation (PLL)	ADAMTS17	000588-9615	September 2010 [10]	Miniature Bull Terrier (MBT)
Episodic falling (EF)	BCAN	001592-9615	January 2012 [11,12]	Cavalier King Charles Spaniel (CKCS)
Congenital keratoconjunctivitis sicca and ichthyosiform dermatosis (Dry eye and curly coat, DE/CC)	FAM83H	001683-9615	January 2012 [13]	Cavalier King Charles Spaniel (CKCS)
Rod cone dysplasia 4 (PRA-rcd4)	C17H2orf71	001575-9615	June 2012 [14]	Gordon Setter (GDN)
				Irish Setter (IRISH)
Spinocerebellar ataxia (SCA)	CAPN1	001820-9615	May 2013 [15]	Parson Russell Terrier (PRT)

Disorders and breeds for which DNA test results were used in this study. OMIA is the Online Mendelian Inheritance in Animals database which provides comprehensive information on the genetics of disorders in animals and from which additional information specific to each disorder may be obtained (http://omia.org/home/).

For each disorder within each breed, the number of test results received for Kennel Club registered dogs per year from 2000 to 2017 was recorded. These data would reveal the usage of specific-disorder DNA tests within particular breeds over time.

For each year from 2000 to 2017 the number of Kennel Club registered dogs born; the number born with an individual DNA test result subsequently recorded; and the number born with individual hereditary results assigned was extracted for each of the eight disorders within each of the eight breeds. From these data, the proportion of the registered population with a known genotype (hereditary status or test result) per year of birth, and the proportion of all those with known genotypes that had a test (rather than hereditary) result was determined. The numbers and proportions of ‘clear’ (wild-type homozygote), ‘carrier’ (heterozygote) and ‘affected’ (mutant homozygote) dogs and the mutation frequency over each year of birth were calculated, from i) only those dogs with test results, and ii) all dogs with known genotypes (both test results and ‘hereditary’ status).

The frequency of the mutant allele, *f(m)*, was calculated as:

$$f(m)=\frac{(2\;n_{m\_hom})+n_{het}}{2\;n_{T}}$$

where *n*_*m_hom*_ is the number of mutant homozygotes (‘affected’), *n*_*het*_ is the number of heterozygotes (‘carriers’) and *n*_*T*_ is the total number with i) a test result or ii) a known genotype.

The frequency of the non-disorder causing (wild type) allele, *f(wt)*, was 1-*f(m)*.

The mutation frequency, *f(m)*, of each disorder within each breed across each year of birth was used to determine any trends. Comparison was made between *f(m)* derived from test results only and from all known genotypes (test results and hereditary status).

The year that the causal mutation of each disorder was published (from Table 1) was denoted *t*. This date approximated to the time that each DNA test was made commercially available and enabled comparisons across breeds and disorders relative to this occurrence. An estimate of *f(m)* in the individual KC breed population prior to the launch of the test was made using data from dogs born in years *t-4* to *t-2* (PRE-TEST_f) as described above. Estimates of *f(m)* in up to three periods subsequent to the test being commercially available, years *t+2* to *t+4* (POST-TEST_f1), years *t+5* to *t+7* (POST-TEST_f2), years *t+8* to *t+10* (POST-TEST_f3) were also calculated, subject to data availability. The standard error (s.e.) of estimates of *f(m)* was calculated as:

$$s.e.=\sqrt{\frac{f(m).f(wt)}{2n}}$$

Using the *f(m)* estimates and s.e., 99.9%, 99% and 95% confidence interval boundaries were computed as estimate±3.291(s.e.), estimate±2.576(s.e.), and estimate±1.96(s.e.) respectively. Non-overlapping estimate confidence intervals of PRE- and POST-TEST *f(m)* estimates within breeds and disorders were used to determine the statistical significance of any differences in *f(m)* estimates since the publication of the mutation and commercial availability of the DNA test (P<0.001, P<0.01 and P<0.05 respectively).

It is noted that estimates of *f(m)* calculated from hereditary status may be subject to intrinsic biases due to the deduction and assignment of genotypes of the progeny of homozygote parents only (the genotypes of progeny of heterozygote parents cannot be definitively deduced). Such biases may yield a reduced estimate of *f(m)* even under random mating, which may be mistaken for a response to selection (although it must be noted that *f(m)* would increase in the cohort of the population with unknown genotypes, remaining static across the population as a whole, and imply the continued production of a consistent proportion of affected individuals per generation). Whether any observed changes in *f(m)* were consistent this phenomenon was tested by analyses undertaken to determine unequivocal evidence of selection (non-random mating). Breeding animals (parents) with known genotypes were identified from the cohort of dogs born in years *t-4* to *t-2* and genotype and allele frequencies were derived. From the parental genotype frequencies, the hereditary progeny genotype frequencies expected under random mating were calculated (expected hereditary clear = *f[AA]*^*2*^, expected hereditary carrier = 2*f[AA]*f[aa], expected hereditary affected = *f[aa]*^*2*^; where *f[AA]*, *f[Aa]* and *f[aa]* denote parental genotype frequencies of wild type/unaffected homozygotes, carrier heterozygotes and affected/mutant homozygotes), and compared to the observed hereditary genotype frequencies of the actual progeny. A chi-squared test of independence was used to determine whether the observed progeny hereditary genotype frequencies were significantly different from those expected under random mating, and so provided evidence of selection (most likely the intentional avoidance of production of affected individuals). Furthermore, the *f(m)* among parents was compared with the *f(m)* of all progeny (which would include potential bias from assignment of hereditary status) and with the *f(m)* from progeny selected to breed (which would not, as it is a comparison of the *f(m)* in consecutive generations of selected breeding animals) to determine the extent of overall changes and change due to selection.

It is also noted that the estimates of *f(m)* calculated using all known genotypes (test results and hereditary status), are derived using less than the entire registered population as genotypes are not available for the whole population. It is conceivable, if unlikely, that this proportion of the registered population with unknown genotypes retains the disease causing mutation at the ‘pre-test’ frequency. Therefore, a ‘worst case scenario’ estimate of current *f(m)* was made allowing for this:

$$\left(p\times POST-TEST\_f_{max}\right)+(\left[1-p\right]\times PRE-TEST\_f)$$

where *p* is the proportion of a registered breed population born in 2017 with known genotypes (tested or hereditary), and POST-TEST_f_max_ is the estimate of *f(m)* made from the most recent post-test period (years *t+2* to *t+4*, *t+5* to *t+7*, or *t+8* to *t+10*).

The influence of population size (as described by average registrations 2000–17) and estimates of PRE-TEST_f on both ‘uptake’ of the test (the proportion of registered dogs with known genotypes born at *t+4*) and proportional decline of estimates of POST-TEST_f1 from PRE-TEST_f were determined by linear regression using R [16]. ‘Uptake’ and ‘decline’ were the dependent y-variables, and ‘population size’ and PRE-TEST_f were fitted independently as explanatory x-variables (a quadratic term for population size was also fitted). Where the x-variable was heteroscedastic (PRE-TEST_f) a weighted least squares regression was used, using weights of 1/variance of the x-variable estimate [variance = *f(m)* x *f(wt)*].

## Results

### Test usage over time

**Fig 1:**
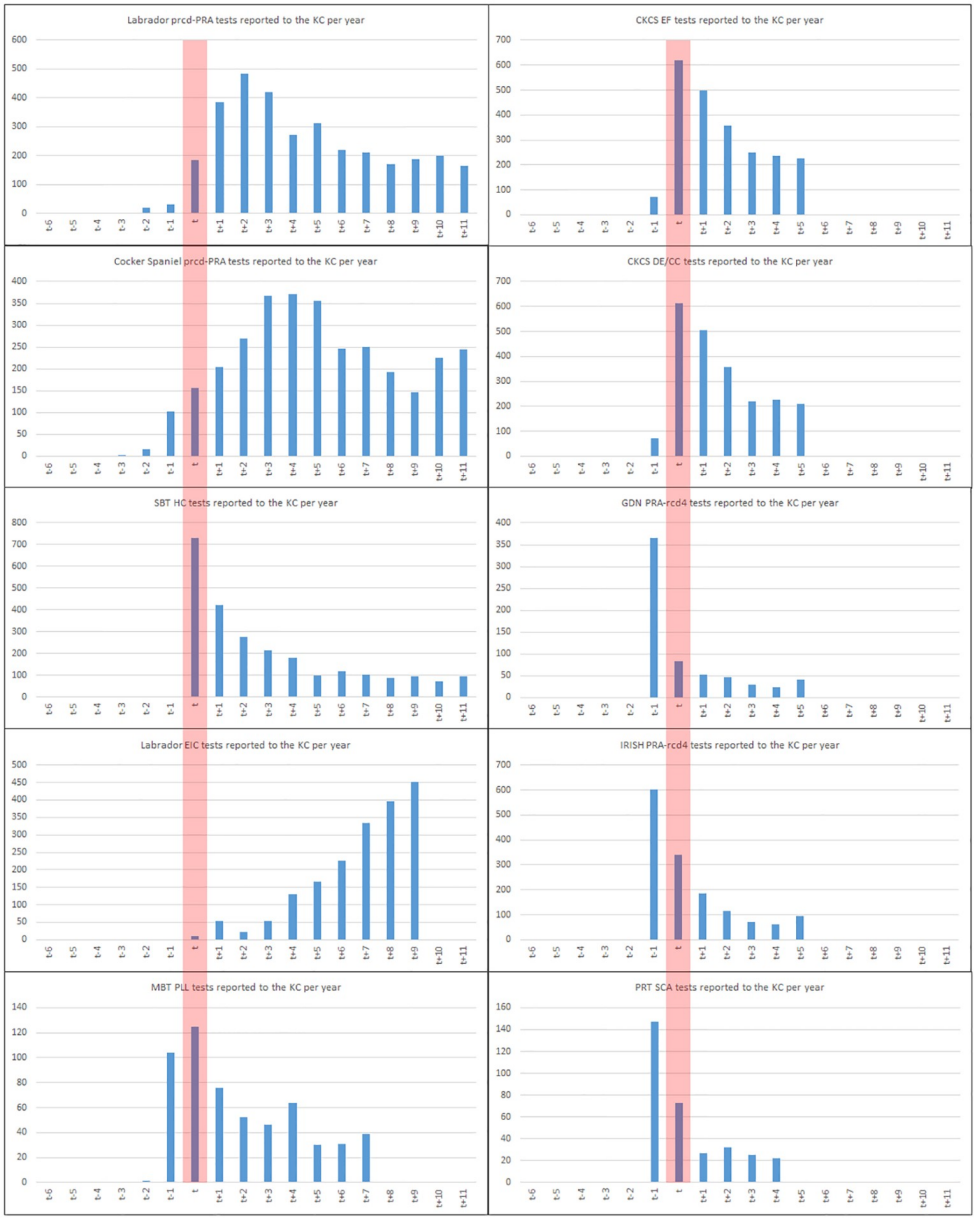
Number of DNA test results per year. Bar charts showing the number of test results reported to the Kennel Club per year for all disorder/breed combinations, where ‘t’ (as indicated by the red bar) is the year that the mutation was published (as in Table 1) thereby standardising the ‘testing profile’ by commercial availability rather than chronological year.

The numbers of test results reported to the Kennel Club per year in most cases showed a discernible peak, approximately around the time that the test became commercially available, followed by a tailing off in most cases (Fig 1, tabular data in S1 Table). In many cases test results were reported to the Kennel Club prior to publication of the mutation (as given in Table 1); for example, Fig 1 shows the largest number of test results for Gordon Setter and Irish Setter reported to the Kennel Club in 2011, when the publication reporting the causal mutation was published in June 2012. In some instances, results from dogs whose samples had been submitted as part of the research that identified the causal mutation may have been recorded by the Kennel Club. However, more likely is the laboratory which identified the causal mutation began offering tests prior to peer review publication of the research identifying the causal mutation. An exception to the ‘peak’ pattern in in tests per year was EIC in Labrador Retriever, where the number of test results reported to the Kennel Club per year is increasing year on year.

### Test results and hereditary status over time

**Fig 2:**
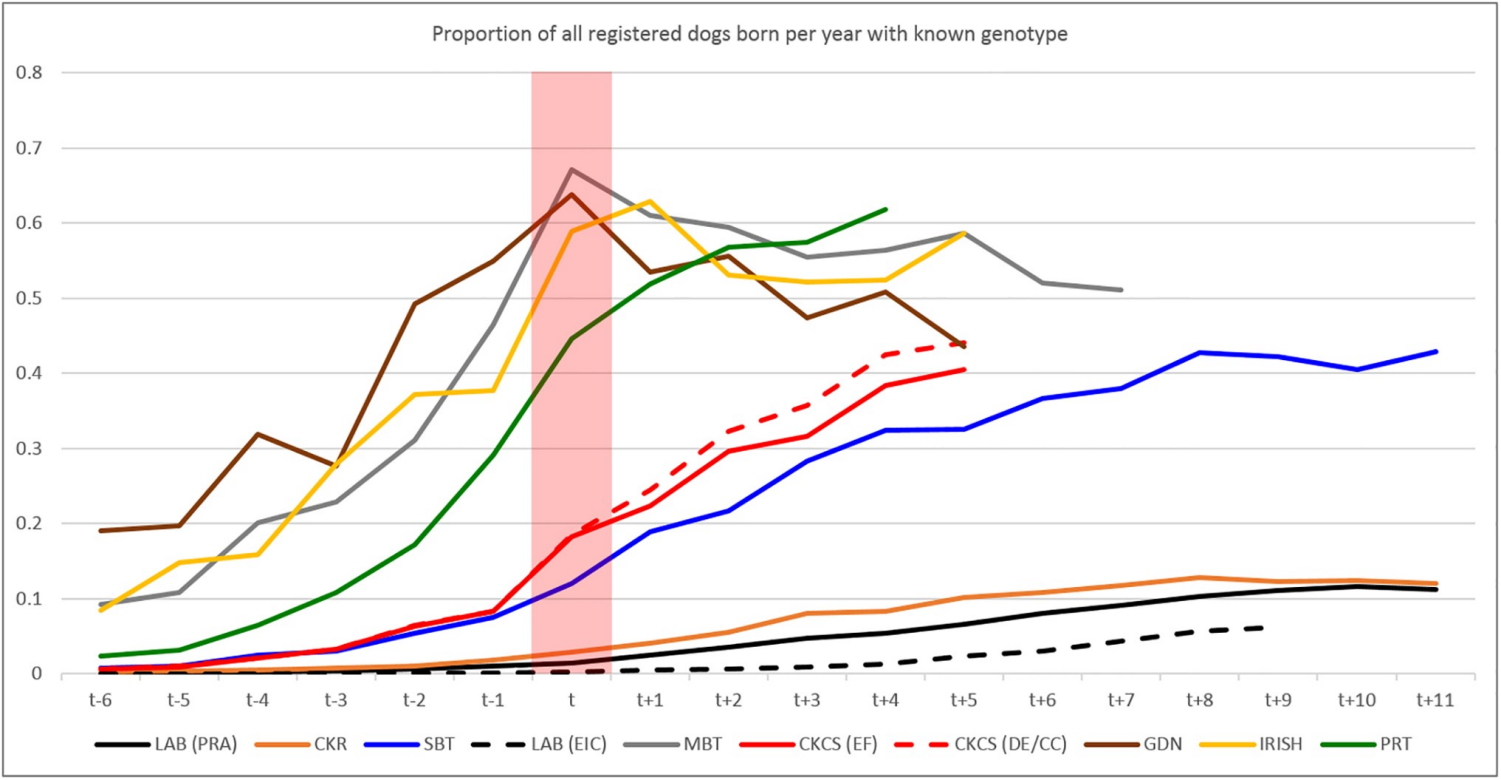
Proportion of dogs with a result. The proportion of Kennel Club registered dogs born per year relative to the year of publication of the mutation (‘t’, as indicated by the red bar) which have a known genotype (either from a test or hereditary status).

**Fig 3:**
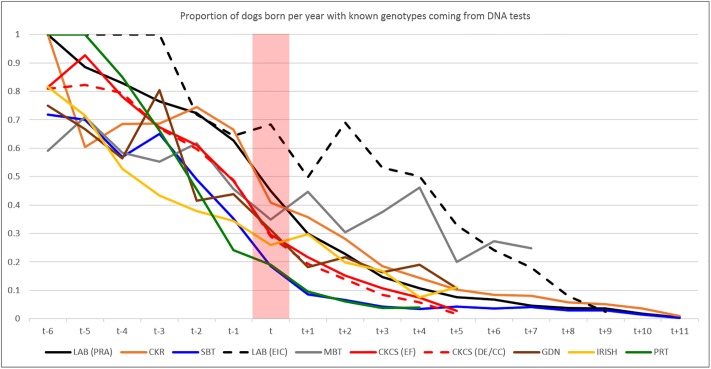
Proportion of all results coming from tests. The proportion of all known genotypes (‘all results’) for Kennel Club registered dogs born per year relative to the publication of the mutation (‘t’, as indicated by the red bar) coming from results of DNA tests reported to the Kennel Club (rather than ‘hereditary status’).

All breeds exhibited an increasing proportion of Kennel Club registered dogs born per year having a known genotype (test result or hereditary status) from t-6 to t+11, while the proportion of results that were test results (rather than hereditary status) declined (Figs 2 and 3, tabular data in S2 Table).

The largest proportion of Kennel Club registered dogs born 4 years after the publication of the disease causing mutation [*t+4*] with known genotypes was in the Parson Russell Terrier for SCA (61.9%) and the smallest in the Labrador Retriever for EIC (1.3%). The rate of increase in the proportion of registered dogs with a known genotype appears notably slower for the Labrador Retriever and Cocker Spaniel compared to other breeds (Fig 2). For the Miniature Bull Terrier, Gordon Setter and Irish Setter the proportion of registered dogs born per year with a known genotype (either from testing or hereditary status) climbs steadily from several years prior to publication of the respective disease causing mutation (*t*), from which point it appears to plateau (Fig 2). All breeds exhibit a declining proportion of known genotypes coming from tests from the years preceding publication of the disease causing mutation (*t)* to the years following (Fig 3). Typically, ‘early’ known genotypes recorded by the Kennel Club are predominantly test results from dogs born several years prior to *t*. However, the proportion of results from tests drops steadily (as hereditary status is applied), comprising less than 20% of all results in all but two cases (Miniature Bull Terrier and Labrador Retriever/EIC) by t+4.

### Prevalence of genotypes over year of birth from all data vs test data only

**Fig 4:**
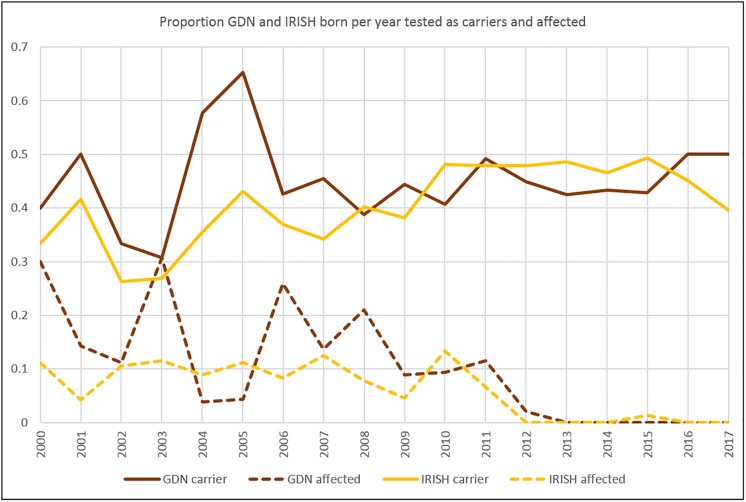
Proportion tested affected and carriers born per year. The proportion of tested Gordon and Irish Setters born per year that tested as carriers and affected. Year ‘t’ for these breeds is 2012.

Tables detailing, for each disorder / DNA test per breed, by year of birth the proportion of clear, carrier and affected individuals using all data (test + hereditary) and using test data only, are shown in S3 Table. Table 2 displays this information for PRA-rcd4 in Gordon Setter and Irish Setter as a typical example. In almost all cases no hereditary affected individuals have been born since test publication (the exception is 3 hereditary affected Cocker Spaniels born in a single year, 2007 or *t+1*). In several cases, there have been no affected individuals (tested or hereditary) born for several years up to 2017, most likely due to a DNA test being available (Staffordshire Bull Terrier, Miniature Bull Terrier, Cavalier King Charles Spaniel DE/CC, Gordon Setter, Parson Russell Terrier). However, in these cases (and in others, e.g. Labrador prcd-PRA, Labrador EIC, Cavalier King Charles Spaniel EF, Irish Setter) the proportion of tested carriers per year of birth does not appear to be declining. Fig 4 shows the proportions of tested ‘carrier’ and ‘affected’ individuals for Gordon Setter and Irish Setter as a typical example. In almost all cases the proportion of carriers from test results alone is higher than (or equal to) that from all data (tests and hereditary results); the only exceptions were 2008 for Gordon Setter, and 2001, 2004, 2005 and 2007 for Irish Setter (Table 2). Furthermore, the proportion of clear dogs from testing data only was always lower than (or equal to) the proportion of clear dogs overall (where hereditary status is included), except 2001 and 2004 for Irish Setter (Table 2).

**Table 2:** Genotype frequencies of registered Gordon and Irish Setters with known genotype born per year.

yob	Gordon Setter						Irish Setter					
	Test results only			All data			Test results only			All data		
	clear	carrier	affected	clear	carrier	affected	clear	carrier	affected	clear	carrier	affected
2000	0.3	0.4	0.3	0.3	0.4	0.3	0.5556	0.3333	0.1111	0.5556	0.3333	0.1111
2001	0.3571	0.5	0.1429	0.3571	0.5	0.1429	0.5417	0.4167	0.0417	0.4063	0.5625	0.0313
2002	0.5556	0.3333	0.1111	0.5556	0.3333	0.1111	0.6316	0.2632	0.1053	0.6316	0.2632	0.1053
2003	0.3846	0.3077	0.3077	0.3846	0.3077	0.3077	0.6154	0.2692	0.1154	0.6774	0.2258	0.0968
2004	0.3846	0.5769	0.0385	0.3846	0.5769	0.0385	0.5556	0.3556	0.0889	0.5510	0.3674	0.0816
2005	0.3044	0.6522	0.0435	0.3044	0.6522	0.0435	0.4583	0.4306	0.1111	0.4607	0.4494	0.0899
2006	0.3148	0.4259	0.2593	0.4722	0.3333	0.1944	0.5476	0.3691	0.0833	0.6311	0.3010	0.0680
2007	0.4091	0.4546	0.1364	0.6061	0.3030	0.0909	0.5333	0.3417	0.1250	0.5595	0.3512	0.0893
2008	0.4032	0.3871	0.2097	0.4364	0.3909	0.1727	0.5196	0.4020	0.0784	0.6580	0.3005	0.0415
2009	0.4667	0.4444	0.0889	0.5	0.4286	0.0714	0.5727	0.3818	0.0455	0.6496	0.2480	0.1024
2010	0.5000	0.4063	0.0938	0.5909	0.3701	0.0390	0.3861	0.4810	0.1329	0.5204	0.4005	0.0791
2011	0.3934	0.4918	0.1148	0.5180	0.4317	0.0504	0.4546	0.4793	0.0661	0.6724	0.3048	0.0228
2012	0.5306	0.4490	0.0204	0.6433	0.3503	0.0064	0.5214	0.4786	0	0.7579	0.2421	0
2013	0.5758	0.4242	0	0.6703	0.3297	0	0.5146	0.4854	0	0.8552	0.1449	0
2014	0.5667	0.4333	0	0.7626	0.2374	0	0.5349	0.4651	0	0.7824	0.2176	0
2015	0.5714	0.4286	0	0.8125	0.1875	0	0.4933	0.4933	0.0133	0.8206	0.1771	0.0022
2016	0.5	0.5	0	0.7759	0.2241	0	0.5484	0.4516	0	0.8519	0.1482	0
2017	0.5	0.5	0	0.9464	0.0536	0	0.6038	0.3962	0	0.9040	0.0960	0

Proportions of PRA-rcd4 clear, carrier and affected Kennel Club registered Gordon and Irish Setters per year born, using all data (test results and hereditary status) and using test results alone.

### Frequency of disease-causing mutation over year of birth

**Fig 5:**
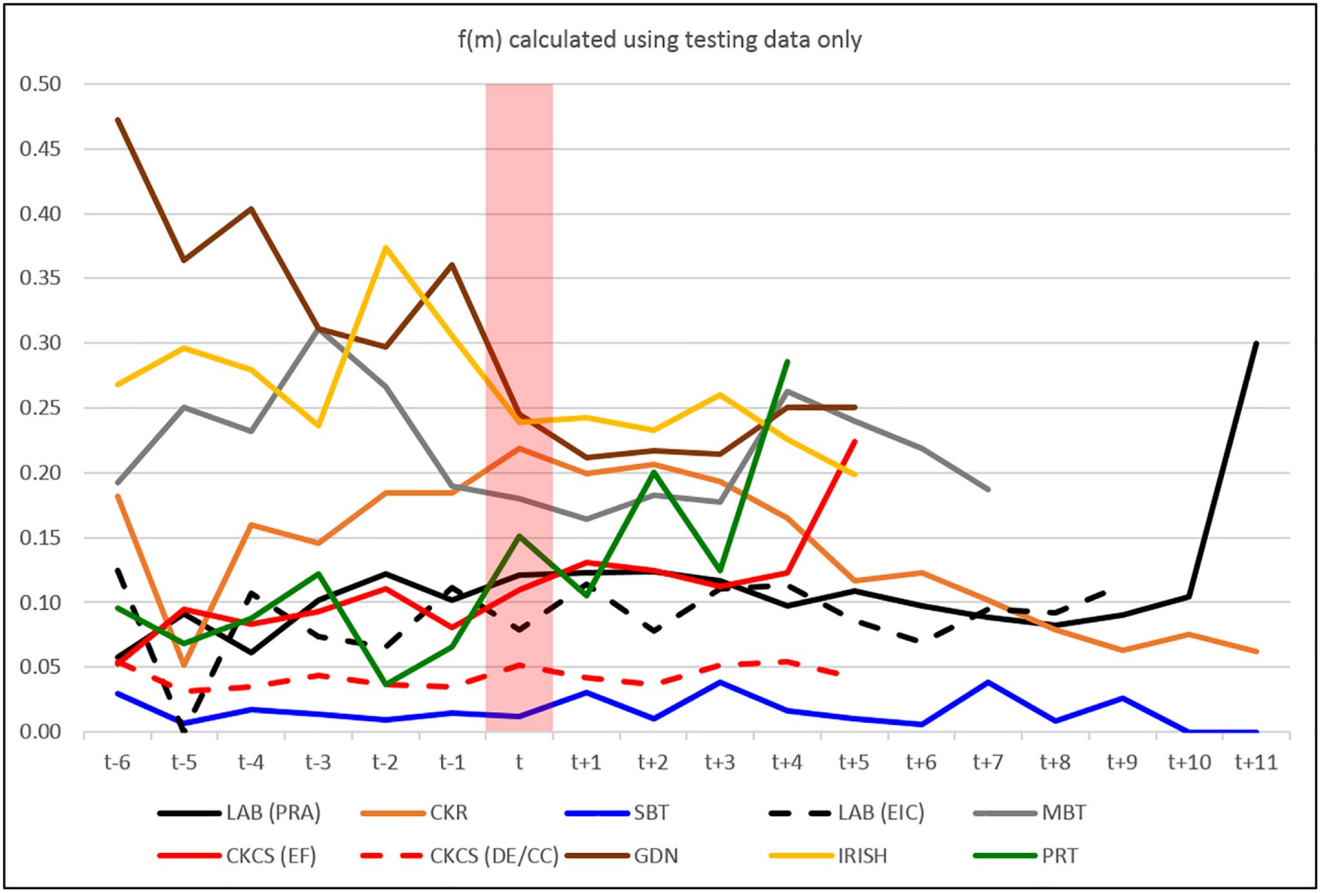
Mutation frequency calculated from test data only. Mutant (disorder-causing) allele frequency, f(m), for each disorder/breed per year of birth relative to the year the mutation was published (t) calculated using test results only.

**Fig 6:**
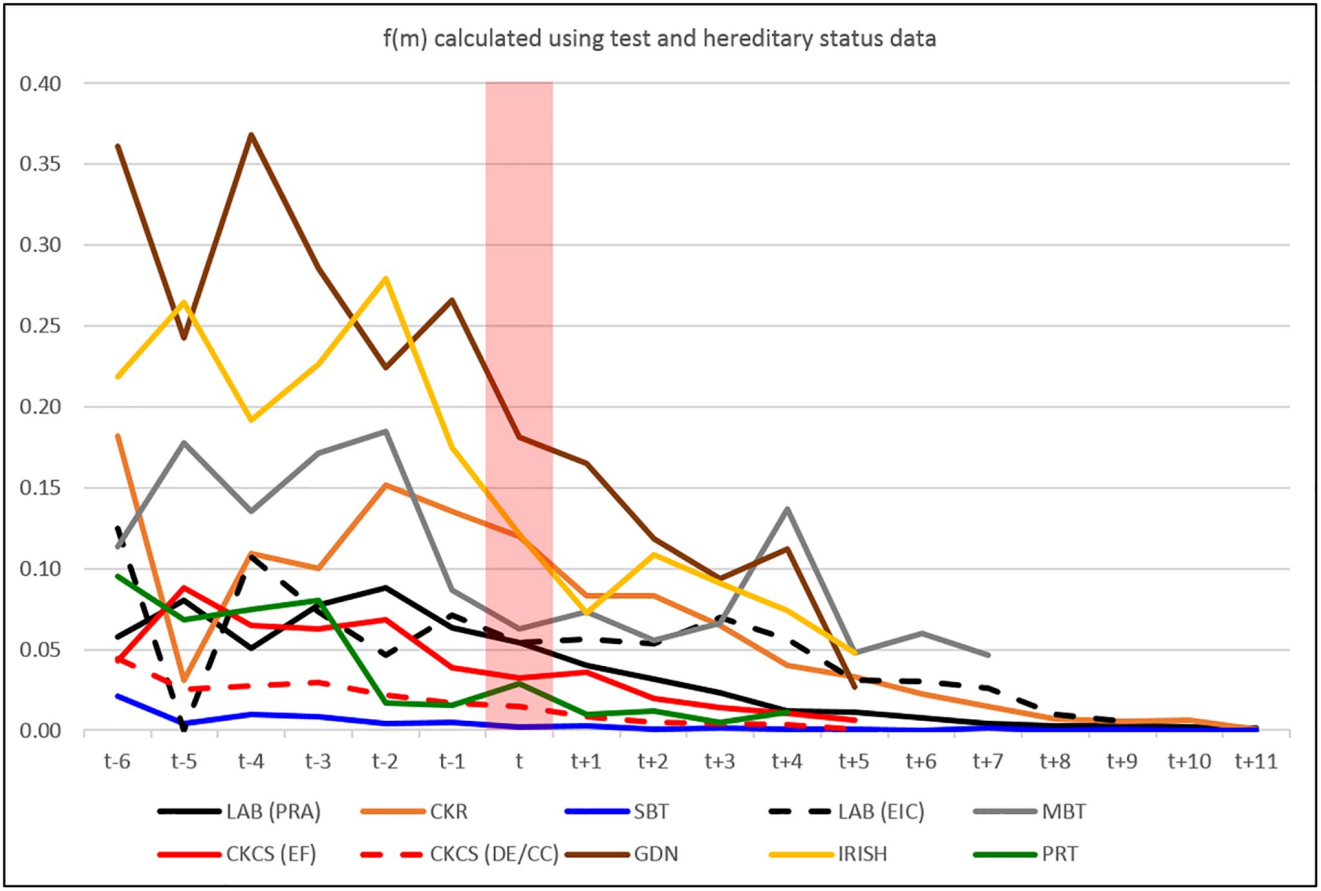
Mutation frequency calculated from all data. Mutant (disorder-causing) allele frequency, f(m), for each disorder/breed per year of birth relative to the year the mutation was published (t) calculated using all known genotype data (test results and hereditary status results).

For each disorder / DNA test per breed, by year of birth the derived frequency of the disease-causing allele, *f(m)*, from test results only are shown in Fig 5. There appeared to be no consistent downward trend across disorders and breeds, even from the levels determined in dogs born prior to publication of the disease causing mutation (*t*) which might be taken as a ‘benchmark’ frequency prior to any test development. In contrast, *f(m)* derived from both test and hereditary results appeared to show a distinct general downward trend across all disorders and breeds (Fig 6). The data in Figs 5 and 6 are given in tabular format in S4 Table.

The results/data described to this point grouped by disorder within breed are given in S5 Table.

### Changes in mutation frequency pre- and post- test availability

To examine the trend in *f(m)* in dogs born in the years since test availability, comparison was made between *f(m)* estimates calculated using all data (test and hereditary) prior to the availability of the test (PRE-TEST_f) and at various periods thereafter (POST-TEST_f1/2/3 where data was available). As noted above, publication appeared in some cases to occur shortly after test availability, so the PRE-TEST_f was taken as the average over years of birth *t-4* to *t-2*. The proportional decline in POST-TEST_f1/2/3 from PRE-TEST_f for each disorder across each breed could then be calculated and compared. These results are displayed in Table 3.

**Table 3:** Estimated mutation frequencies pre- and post-test availability.

Disorder	prcd-PRA		HC	EIC	PLL	EF	DE/CC	PRA-rcd4		SCA
Breed	LAB	CKR	SBT	LAB	MBT	CKCS	CKCS	GDN	IRISH	PRT
pre-test										
n	533	356	1274	80	188	1010	1015	320	864	199
f(m)	0.0779	0.1264	0.0067	0.0688	0.1676	0.0658	0.0251	0.2844	0.2442	0.0503
s.e.	0.0082	0.0125	0.0016	0.0200	0.0193	0.0055	0.0035	0.0178	0.0103	0.0110
post-test1										
n	5928	4981	7450	1077	391	4362	4839	383	1283	640
f(m)	0.0209	0.0600	0.0009	0.0604	0.0857	0.0147	0.0041	0.1084	0.0916	0.0094
s.e.	0.0013	0.0024	0.0003	0.0051	0.0100	0.0013	0.0007	0.0112	0.0057	0.0027
% decline	73.13%	52.51%	85.92%	12.21%	48.87%	77.72%	83.55%	61.90%	62.50%	81.34%
Diff to pre-test	***	***	**	ns	**	***	***	***	***	**
post-test2										
n	8714	7607	6807	3316	339	1376	1499	112	479	
f(m)	0.0076	0.0232	0.0007	0.0286	0.0516	0.0062	0.0007	0.0268	0.0480	
s.e.	0.0007	0.0012	0.0002	0.0020	0.0085	0.0015	0.0005	0.0108	0.0069	
% decline	90.27%	81.64%	88.99%	58.33%	69.19%	90.62%	97.34%	90.58%	80.34%	
Diff to pre-test	***	***	**	ns	***	***	***	***	***	
Diff to post-test1	***	***	ns	***	ns	**	**	***	***	
post-test3										
n	11093	8362	5825	4088						
f(m)	0.0027	0.0062	0.0003	0.0076						
s.e.	0.0003	0.0006	0.0002	0.0010						
% decline	96.53%	95.08%	94.85%	88.97%						
Diff to pre-test	***	***	***	**						
Diff to post-test1	***	***	ns	***						
Diff to post-test2	***	***	ns	***						

The number of individuals with known genotype, estimated mutant (disorder causing) allele frequency, *f(m)*, and standard error (s.e.) for each disorder/breed within pre- and post- test periods 1, 2 and 3 (t-4 to t-2, t+2 to t+4, t+5 to t+7 and t+8 to t+10 respectively). Percentage decline from pre-test estimates for post-test estimates are given. Statistical significance of the difference between estimates from confidence interval testing is indicated as:

‘***’ P<0.001,

‘**’ P<0.01,

‘*’ P<0.05,

‘ns’ P>0.05.

From Table 3 PRE-TEST_f ranged widely from 0.67% (HC in Staffordshire Bull Terrier) to 28.44% (PRA-rcd4 in Gordon Setter). POST-TEST_f1, which was available for all disorders/breeds, ranged from 0.09% (HC in Staffordshire Bull Terrier) to 10.84% (PRA-rcd4 in Gordon Setter), representing an 85.92% and 61.90% decline respectively. A decline in *f(m)* of over 80% in the same time period was observed in 3 disorders/breeds (HC, Staffordshire Bull Terrier; DE/CC, Cavalier King Charles Spaniel; SCA, Parson Russell Terrier). The smallest decline was observed for the *f(m)* for EIC in Labrador Retrievers (12.21%). Proportional declines in POST-TEST_f2 ranged from 58.33% (EIC, Labrador Retriever) to 97.34% (DE/CC, Cavalier King Charles Spaniel), although this latter figure was based on one year’s data. Proportional declines in POST-TEST_f3 were only available for 4 disorder/breed combinations, and ranged from 88.97% (EIC, Labrador Retriever) to 96.53% (prcd-PRA, Labrador Retriever). All disorder causing mutations within all breeds showed a significant decline in frequency in at least one of the POST-TEST estimates from PRE-TEST_f estimates. For EIC in Labrador Retrievers only POST-TEST_f3 was significantly different to PRE-TEST_f, although differences between POST-TEST_f1, POST-TEST_f2 and POST-TEST_f3 were all significant. In both the Miniature Bull Terrier (PLL) and the Staffordshire Bull Terrier (HC), while POST-TEST_f1 were significantly lower than PRE-TEST_f, the differences between POST-TEST estimates of *f(m)* were not significant.

In six of the ten disorder/breed combinations (prcd-PRA, Labrador Retriever and Cocker Spaniel; PLL, Miniature Bull Terrier; EF, Cavalier King Charles Spaniel; PRA-rcd4, Gordon Setter and Irish Setter) there was a highly significant difference (P<0.1x10^-4^) between the observed progeny hereditary genotype frequencies and those expected from random mating of parents (born *t-2 to t-4* years) with known genotypes, providing unequivocal evidence of selection. In four disorder/breed combinations (HC, Staffordshire Bull Terrier; EIC, Labrador Retriever; DE/CC, Cavalier King Charles Spaniel; and SCA, Parson Russell Terrier) the analysis was not possible due to a complete absence of affected parents used for breeding, meaning no ‘hereditary affected’ or ‘hereditary carrier’ genotypes would occur even under random mating. Table 4 shows the *f(m)* of parents (born *t-2* to *t-4* years) with known genotype, *f(m)* of their breeding progeny, and the overall change and percentage decline representative of selection, in all disorder/breed combinations. The declines range from 27.0% (Parson Russell Terrier) to 83.9% (Staffordshire Bull Terrier) of parental *f(m)*. Also included in Table 4 is the *f(m)* of all progeny, which will include the potential inherent bias due to assignment of hereditary status, and the decline in this from parental *f(m)*. The proportion of the decline in *f(m)* from all progeny accounted for by the decline in *f(m)* from breeding progeny only may provide insight into the extent of bias introduced by the assignment of hereditary status per breed. In all disorder/breed combinations there appeared to be some bias introduced (i.e. the decline in *f(m)* of breeding progeny was smaller than the decline in *f(m)* from all progeny), except for PLL in Miniature Bull Terriers. However, the decline observed from breeding progeny accounted for a substantial proportion of the decline observed from all progeny (41% to 96%, and exceeding 60% in seven of the remaining disorder/breed combinations), indicating that change due to selection was most often predominant.

**Table 4:** Decline in mutation frequency of all progeny, and breeding progeny only, compared to parental mutation frequency.

Disorder	prcd-PRA		HC	EIC	PLL	EF	DE/CC	PRA-rcd4		SCA
Breed	LAB	CKR	SBT	LAB	MBT	CKCS	CKCS	GDN	IRISH	PRT
f(m) parents	0.0891	0.1486	0.0087	0.0797	0.1875	0.0794	0.0354	0.2917	0.2130	0.0575
f(m) breeding progeny	0.0342	0.0926	0.0014	0.0366	0.0652	0.0396	0.0163	0.1615	0.1544	0.0420
change (selection)	-0.0549	-0.0561	-0.0073	-0.0431	-0.1223	-0.0398	-0.0191	-0.1301	-0.0586	-0.0155
% decline	61.62%	37.69%	83.91%	54.08%	65.23%	50.13%	53.95%	44.63%	27.51%	26.96%
f(m) all progeny	0.0195	0.0526	0.0012	0.0101	0.0714	0.0201	0.0050	0.1361	0.1319	0.0201
change (selection + bias)	-0.0696	-0.0960	-0.0076	-0.0696	-0.1161	-0.0593	-0.0304	-0.1556	-0.0811	-0.0374
%decline	78.11%	64.60%	86.21%	87.33%	61.92%	74.69%	85.88%	53.34%	38.08%	65.04%
% change due to selection only	78.88%	58.44%	96.05%	61.93%	105.34%	67.12%	62.83%	83.61%	72.26%	41.44%

For each disorder/breed the *f(m)* of breeding individuals born at *t-2* to *t-4* years (parents) with known genotype, the *f(m)* of breeding progeny of parents and the *f(m)* of all progeny of parents. Percentage decline from parental *f(m)*, and the proportion of change in *f(m)* from all progeny (selection + bias) that is accounted for by the change in *f(m)* from breeding progeny only (selection) is given.

Table 5 shows the ‘worst case scenario’ *f(m)* with the proportion of each breed with unknown genotypes assumed to have *f(m)* of PRE-TEST_f. The biggest impact in terms of altered decline was in Labrador Retriever and Cocker Spaniel (only 5.47% and 10.89% compared to 88.97% to 96.53% for Labrador Retriever EIC and prcd-PRA respectively, and 11.38% compared to 95.08% for Cocker Spaniel). However, for other breeds the ‘adjusted’ or ‘worst case scenario’ decline in *f(m)* remains sizable even if, as expected, less than the decline estimated in Table 3.

**Table 5:** Decline in mutation frequency assuming mutation frequency at pre-test levels among dogs of unknown genotype.

Disorder	prcd-PRA		HC	EIC	PLL	EF	DE/CC	PRA-rcd4		SCA
Breed	LAB	CKR	SBT	LAB	MBT	CKCS	CKCS	GDN	IRISH	PRT
p (2017)	0.1128	0.1196	0.4291	0.0615	0.5105	0.4047	0.4409	0.4358	0.5870	0.6186
pre test f(m)	0.0779	0.1264	0.0067	0.0688	0.1676	0.0658	0.0251	0.2844	0.2442	0.0503
max post-test f(m)	0.0027	0.0062	0.0003	0.0076	0.0516	0.0062	0.0041	0.0268	0.0480	0.0094
adjusted f(m)	0.0694	0.1120	0.0040	0.0650	0.1084	0.0417	0.0159	0.1721	0.1290	0.0250
decline %	10.89%	11.38%	40.70%	5.47%	35.32%	36.67%	36.83%	39.48%	47.16%	50.32%

‘Adjusted’ *f(m)* assuming registered dogs without results have *f(m)* at pre-test levels. ‘p (2017)’ is the proportion of registered dogs born in 2017 with a result, ‘adjusted *f(m)*’ is (p * max post-test *f(m)*) + ([1-p] * PRE-TEST_f), and decline is the adjusted *f(m)* / PRE-TEST_f.

### Influences on uptake of test and decline in mutation frequency

The influence of PRE-TEST_f and breed population size (mean number of registrations 2000–17) on ‘uptake’ of the test (as the proportion of registered population with a known genotype at *t+4*) and the decline in *f(m)* (percent decline of POST-TEST_f1 from PRE-TEST_f) across disorder / breed combinations were ascertained. Data is shown in Table 6.

**Table 6:** Breed population size, pre-test mutation frequency, test ‘uptake’, and decline in mutation frequency per breed/disease.

Disorder	Breed	mean regs	f(m)	% decline	uptake—% reg
		2000–17	pre-test	post-test1	with result t+4
prcd-PRA	LAB	39139.2	0.0779	73.13%	0.0546
	CKR	19866.6	0.1264	52.51%	0.0825
HC	SBT	8763	0.0067	85.92%	0.3239
EIC	LAB	39139.2	0.0688	12.21%	0.0129
PLL	MBT	232.17	0.1676	48.87%	0.5639
EF	CKCS	8245.5	0.0658	77.72%	0.3840
DE/CC	CKCS	8245.5	0.0251	83.55%	0.4246
PRA-rcd4	GDN	304.5	0.2844	61.90%	0.5088
	IRISH	1063.5	0.2442	62.50%	0.5246
SCA	PRT	621.1	0.0503	81.34%	0.6186

Mean registrations (2000–17) for each breed, PRE-TEST_f, percent decline in f(m) of POST-TEST_f1, and test ‘uptake’ as proportion of registered dogs with a result at t+4, for each disorder/breed combination.

**Fig 7:**
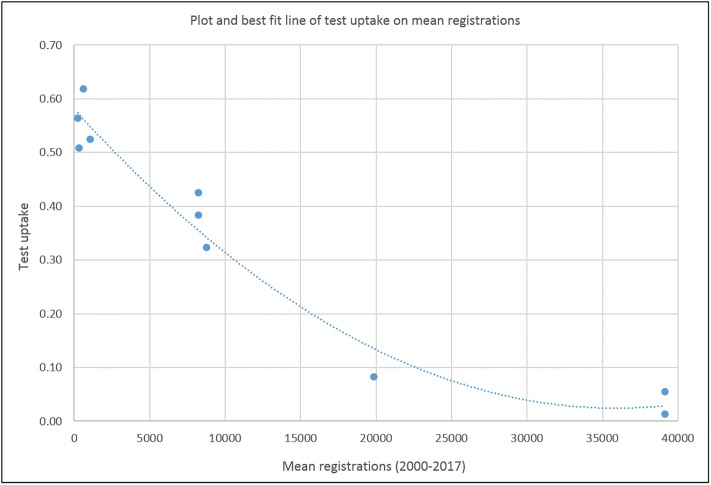
Plot of test uptake on breed population size. Plot and best fit line of test uptake (proportion registered dogs with result at t+4) on breed population size (mean registrations, 2000–17).

There was a strong negative relationship between test uptake and breed population size (Fig 7). Regression of test uptake on breed population size yielded an R-squared value of 0.9611 and an F-statistic of 86.50 (p<0.001), implying that population size accounted for much of the variation in test uptake and that this was significantly greater than zero. None of the other regressions revealed R-squared values that were either large or significantly different to zero (S1–S3 Figs).

## Discussion

This study has demonstrated general and sizable declines in the frequencies of disease-causing mutations in Kennel Club registered breed populations following the availability of commercial DNA tests. This observed trend implies that DNA testing is being embraced by breeders, and suggests that they are using the results to influence their breeding strategies and select against the disease causing mutations.

The scale and significance of the decline in *f(m)* from pre-test levels in the populations examined in this study was notable, with a decline in *f(m)* of over 45% in dogs born two to four years following publication of the causal mutation for all disorders in all breeds, except EIC in the Labrador Retriever (as shown in Table 3). In dogs born eight to ten years after publication of the causal mutation, the *f(m)* had dropped by nearly 90% or more from pre-test levels (i.e. the *f(m)* was at 10% or less of pre-test levels) for the four diseases for which there were data (including EIC in Labrador Retrievers). The results presented show that the observed declines are in the most part due to selection being applied against the disease causing mutations in Kennel Club registered breed populations.

Previous analyses of changes in causal mutation frequency have been made from data unavoidably restricted to dogs presenting for testing. These analyses have, therefore, been hampered by non-availability of ‘hereditary’ status data on dogs whose recent ancestors had themselves tested ‘clear’, and whose breeders were utilising stock they knew to be free from the mutation. This study enabled comparative analysis of changes under these same conditions (i.e. when the sample was restricted to only those dogs that been tested themselves) and revealed a relatively consistent proportion of heterozygote ‘carriers’ over time and no generally observable decline in *f(m)*, despite a drop in the proportion of ‘affected’ dogs. In contrast, by including ‘hereditary’ data, this study was able to utilise a sample that better reflected the likely true proportion of each breed population with a known genotype, which included that proportion which has already undergone selection against the mutation, and so yields an estimate of *f(m)* more representative of the entire population. When this section of data is included, the general and sizeable decline in the frequency of disease causing mutation is observed.

This study was able to include data on genotypes known via hereditary status and so eliminate some of the bias in estimating *f(m)* in populations encountered using test result data alone. However, the question over the extent of bias in our samples, and what effect this may have, remains given both the inherent bias in only being able to determine and assign ‘hereditary’ genotypes in progeny of homozygote parents, and that there exists a further proportion of registered populations from which genotype data are missing. We sought to account for this by two means; firstly, by demonstrating that selection against the disease causing mutation was occurring and was the predominant cause of detected changes in *f(m)*. Secondly, we derived a ‘worst case scenario’ estimate of *f(m)* within each breed, by assuming that, in the proportion of the registered breed population for which genotypes were unknown, the *f(m)* was at estimated pre-test levels. Even under this assumption there were sizable declines in *f(m)*, ranging between 5% and 50% of pre-test levels. However, this is likely to be an overly-stringent assumption since, if true, it implies that, in the proportion of the population with no data (often in the region of >50% of registered dogs born per year), there is a disease causing mutation at a frequency high enough to cause a disease at a prevalence which initially attracted the attention of owners, breeders, veterinarians and researchers. Thus, breeders and veterinary surgeons are likely to be well aware of the issue within the breed, and the availability of a DNA test. This principle also applies to the cohort of the population with non-determinable hereditary genotypes in which, unless there is selection against the mutation, *f(m)* will increase (as it declines in among animals with ‘hereditary status’) resulting in continued production of affected individuals each generation. It seems unlikely that a disorder for which there is an easily obtainable definitive DNA test would continue to be tolerated in a large proportion of a population under these circumstances. Thus, we may reasonably surmise that the disorder does not exist at the same prevalence in this section or cohort of the breed, and so the causal mutation in the cohort with unknown genotypes is at a lower frequency than the derived pre-test frequency. However, the same rationale could be applied to our estimation of pre-test *f(m)*, prior to location of the causal mutation (i.e. that it was at varying frequencies in particular sub-sections of the breed), and could imply that our pre-test *f(m)* is an overestimate of the frequency across the entirety of each breed. This bias may have been exacerbated by recruitment of affected dogs and their relatives during research into the location of the causal mutation. Therefore, the true extent of decline in *f(m)* from true pre-test levels is likely to rest somewhere between our worst case scenario and reported estimates.

The pattern of test results per year (Fig 1), proportion of registered population per year of birth with a known genotype (Fig 2) and the proportion of all known genotypes in registered populations per year of birth that were test results (Fig 3) reveal some clues as to how tests are used by breeders (both in the development stage and once commercially available). Initially, in all cases the number of test results peaks at, or near, the time that the causal mutation was published (the only exception being EIC in the Labrador Retriever where the number of test results per year continues to increase, Fig 1). This is likely to be a consequence of the research effort leading to identification of the causal mutation, a process in which breeders often actively participate via providing DNA samples from dogs verified as affected or unaffected by veterinary surgeons (e.g. [11], [13], [14]). If the disease has been particularly prevalent in a breed, then the successful culmination of the research is likely to be met with enthusiasm and some breeders may elect to genotype all their dogs immediately. Despite the number of test results recorded per year subsequently beginning to tail off, the proportion of the registered population born per year with a known genotype continues to grow (Fig 2). Comparison with Fig 3 shows that this growth is mainly due to the increase in numbers of dogs with a ‘hereditary status’; while the proportion of registered populations born four years after publication of the mutation with a known genotype is continuing to grow, the proportion of these comprising test results continues to dwindle (less than 20% in all except EIC for Labrador Retrievers and PLL for Miniature Bull Terriers). It appears that once a DNA test is available there is often a ‘spike’ in uptake, which has the almost immediate effect of nearly eliminating the production of ‘affected’ puppies (Table 2, Fig 4, S3 Table). Subsequently, breeding stock appears eventually to be selected on the basis of candidates being ‘clear’ (homozygous for the wild type allele) and breeders then use such candidates to produce subsequent generations known to be free from the disease causing mutation. It is important to note that DNA tests for autosomal recessive disorders enable breeders to safely utilise ‘carriers’ (heterozygotes) in their breeding strategies without the risk of producing affected puppies, a strategy which may help to avoid a sudden loss of genetic diversity due to intense selection against the disease causing mutation. Such strategies will become increasingly important as the number of selection criteria with respect to health (DNA tests for disease and phenotypes from screening programmes) continues to increase in most breeds.

There does appear to be some variation in the rate of increase in the proportion of a registered population having a known genotype (Fig 2) associated with the numeric size of the breed. The slowest rate of increase occurs in the two numerically large breeds in this study (Labrador Retriever, with a mean of 39,139 registered dogs born per year 2000–17; Cocker Spaniel, mean = 19,867), and the proportion of registered dogs born per year with a known genotype remains disappointingly low in some of the numerically larger breeds. Conversely, numerically smaller breeds (with a few hundred individuals born per year; Gordon and Irish Setters, Parson Russell Terrier and Miniature Bull Terrier) exhibit a notably higher rate of increase in proportion with a known genotype (although in some this appeared to ‘plateau’ at around the time of publication of the causal mutation). The Cavalier King Charles Spaniel (mean = 8,246) and the Staffordshire Bull Terrier (mean = 8,763) appear to be grouped together between the numerically large and small breeds. Regression of ‘uptake’ (proportion of individuals born in *t+4* with a known genotype) on population size showed a sizable and highly significant influence, the only one detected on either response variables, ‘uptake’ or decline in *f(m)*. A reason for this may be that numerically smaller breeds are perhaps more likely to have a single, cohesive ‘community’ of breeders, which may result in a more unified approach to tackling the disease. Conversely the Labrador Retriever and Cocker Spaniel breeds may have significant population substructure, both in terms of function (working/show/pet) and in geography, and the much greater number of stakeholders might result in a more disparate approach. This, combined with the small proportion of the entire registered breed with known genotypes, could imply some bias in the data for these breeds. The ‘plateau’ in the proportion of registered individuals born per year with known genotypes in some breeds is concerning, particularly since the mutation frequency and proportion of carrier individuals from testing alone appears not to generally be falling. This lack of decline in mutation and carrier frequency from test result data could imply that breeders are following the strategy suggested previously, namely to safely use carriers for breeding (to known clear dogs) and test the progeny to determine genotypes. However, it could also potentially be some evidence that the mutation frequency in the unknown cohort is not very different to the derived pre-test frequency, counter to the speculation above (of varying/lower *f(m)* in some sections or cohorts of the breed). Perhaps in numerically smaller breeds the existence of [semi] distinct subpopulations (and variation in *f(m)* across them) is less likely. Nevertheless, there may still be some ‘structure’ to the breed which influences gene flow (akin to the ‘nucleus’, ‘multiplier’, and ‘commercial’ herds in some livestock populations), which may explain the persistence of disease-causing mutations in the smaller number of individuals born and undergoing testing several years after the disease causing-mutation was published. Furthermore, the reduction in prevalence as a consequence of selection could engender a belief that this disease is no longer a prominent welfare issue for the breed. A greater understanding of the structure of dog breed populations and how this influences gene flow would be useful to better understand this area.

This study includes breed-specific data pertaining to eight distinct autosomal recessive disorders. To include data for all inherited disorders for which DNA testing data are available would have been prohibitively lengthy, so a subset of disorders was selected that exhibited a range of initial *f(m)*. We selected tests within breeds of varying numerical size, from the relatively rare (e.g. Miniature Bull Terrier with a mean of 232 dogs registered 2000–17; and Gordon Setter with a mean of 305 dogs registered per year) to the very popular (Cocker Spaniel with a mean of 19,867 dogs registered per year 2000–17; and Labrador Retriever with a mean of 39,139 dogs registered per year). Two of the disorders included in the study, EIC and prcd-PRA, affect the Labrador Retriever, which enabled comparison of the response to each within a single breed. From Fig 2 it can be seen that the rate of increase in proportion of registered population with a known genotype for prcd-PRA is higher than for EIC, suggesting a delayed uptake in testing for EIC (although it is still increasing). Furthermore, data from Fig 6 and Table 3 show that the decline in *f(m)* from pre-testing levels (the posited ‘response’ to selection) was, initially, greater for prcd-PRA than EIC. Although significant declines in estimates of POST-TEST_f1, POST-TEST_f2 and POST-TEST_f3, and POST-TEST_f3 being significantly smaller than PRE-TEST_f, reveal a general reduction in *f(m)* for EIC (despite differences in POST-TEST_f1/2 estimates not being significantly different to PRE-TEST_f, probably because a small sample size to determine PRE-TEST_f yielded a wide confidence interval), this was slower than for prcd-PRA. Possible explanations might include that EIC is not considered by breeders as either sufficiently debilitating or prevalent to represent as significant a welfare burden as prcd-PRA to the breed. Progressive rod-cone degeneration (prcd-PRA) is an irreversible and ultimately blinding condition that cannot be treated [17] and the causal mutation occurs in many breeds [7]. Exercise induced collapse (EIC) is manifested by muscle weakness, incoordination and collapse after intense exercise [9]. While the collapse has the potential to be life-threatening, in most cases the clinical signs, despite being distressing for the dog and owner, are temporary and can be effectively avoided through management. Thus, it may be that the prognosis of each disease has meant that tackling prcd-PRA has been prioritised over EIC. It is also likely that genetic testing for EIC was delayed in its adoption and is now increasing in the UK because this test became more easily accessible after US patent law changes in 2012 meant submission of samples internationally to the USA was no longer required. In addition, differences in prevalence could also influence prioritisation. The pre-test *f(m)* of prcd-PRA and EIC in the Labrador Retriever was similar (0.0779 vs 0.0688), meaning the cause of any variation in prevalence of the disorders was not likely to be due to differential prevalence of the disease-causing mutations. However, differences in *f(m)* confounded with population substructure (more likely to exist in numerically larger breeds) could manifest as differences in observed prevalence between prcd-PRA and EIC among sub-groups. Furthermore, differences in exercise intensity and patterns among sub-groups could have a further influence; pet Labrador Retrievers are likely to be much more sedentary than their working cousins, and so perhaps less likely to attain the threshold of exertion necessary to induce a collapse. That many Labrador Retrievers might never experience sufficiently intense exercise to induce an episode of collapse, regardless of their genotype, may mean the disorder is not perceived to be sufficiently prevalent to warrant genetic testing and/or selection against the disorder.

Although this study has shown the value of definitively inferred genotypes (via ‘hereditary status’) in providing more complete information on the population (even given the inherent bias discussed previously), there are three caveats over the veracity of this type of data. Firstly, and least likely, is the possibility that exactly the same mutation occurs *de novo* (although as stated in the introduction, the probability of this is very, very small). Secondly, there is the possibility of an erroneous genotype being assigned to an individual due to failures in testing protocols, or that the incorrect dog is named as the sample donor (although the motivation for intentional deceit is low and such errors will usually be discovered). Thirdly, and probably most common, there is the issue of incorrect recording of parentage, which may result in the incorrect assignment of ‘hereditary’ status. Again, although errors in genotypes due to such failures are likely to be discovered eventually, they may possibly entail the unwitting production of affected individuals. Given that DNA tests provide a ready, and relatively affordable, means to ensure that affected individuals are never born again, and that assignment hereditary status is not currently conditional on DNA verified parentage, it would be advisable for breeders to re-test ‘hereditary clear’ animals every few generations or so to ensure such genotypes are correct. There are some potential biases to consider in the test result data too, including clustering of testing among closely related dogs and potential preferential reporting of clear results in the small number of data reported directly from owners to the Kennel Club.

In conclusion, this study has demonstrated a general and sizeable decline in the frequencies of several disease-causing mutations within several breeds following the commercial availability of DNA tests. This decline was only observable using data which included the ‘hereditary status’ of dogs, whose ancestors had undergone DNA testing and whose genotypes were predictable. Data from DNA testing alone, while often revealing the near immediate and total cessation of production of affected individuals, did not demonstrate a general reduction in either the mutation frequency or the proportion of carrier animals. It is thought likely that the structure of breeding populations, which influences gene flow, may have an effect on these parameters. It is hoped that results from this and similar analyses may provide evidence on which to base advice and guidelines for the selection against disease causing mutations in registered dog populations.

## Supporting information

S1 Table Number of tests per year. The numbers of tests per year for each disorder / breed combination; progressive rod-cone degeneration (prcd-PRA), early onset [hereditary] cataract (HC), exercise-induced collapse (EIC), lens luxation (PLL), episodic falling (EF), congenital keratoconjunctivitis sicca and ichthyosiform dermatosis (Dry eye and curly coat, DE/CC), rod cone dysplasia 4 (PRA-rcd4), spinocerebellar ataxia (SCA); Labrador Retriever (LAB), Cocker spaniel (CKR), Staffordshire Bull Terrier (SBT), Miniature Bull Terrier (MBT), Cavalier King Charles Spaniel (CKCS), Gordon Setter (GDN), Irish Setter (IRISH), Parson Russell Terrier (PRT). Grey shading denotes the year the causal mutation was published ('t') as listed in Table 1.
(DOCX)

S2 Table Proportion of registered dogs with a known genotype and source. The number of Kennel Club registered dogs born per year (‘nb’) which have a known genotype (‘result’ [‘nr’]—either from a test or hereditary status), and those which are test results (‘nt’). Proportion of those born with a known genotype (‘nr/nb’) and of those with known genotypes that have a test result (‘nt/nr’). Grey shading denotes the year the causal mutation was published ('t') as listed in Table 1. Data given for each disorder / breed combination; progressive rod-cone degeneration (prcd-PRA), early onset [hereditary] cataract (HC), exercise-induced collapse (EIC), lens luxation (PLL), episodic falling (EF), congenital keratoconjunctivitis sicca and ichthyosiform dermatosis (Dry eye and curly coat, DE/CC), rod cone dysplasia 4 (PRA-rcd4), spinocerebellar ataxia (SCA); Labrador Retriever (LAB), Cocker spaniel (CKR), Staffordshire Bull Terrier (SBT), Miniature Bull Terrier (MBT), Cavalier King Charles Spaniel (CKCS), Gordon Setter (GDN), Irish Setter (IRISH), Parson Russell Terrier (PRT).
(XLSX)

S3 Table Genotype proportions from tested and all data by year of birth. Table detailing, by year of birth, the proportion of clear, carrier and affected individuals using all data (test + hereditary, ‘overall’) and using test data only (‘tested’). Grey shading denotes the year the causal mutation was published ('t') as listed in Table 1. Data given for each disorder / DNA test per breed; progressive rod-cone degeneration (prcd-PRA), early onset [hereditary] cataract (HC), exercise-induced collapse (EIC), lens luxation (PLL), episodic falling (EF), congenital keratoconjunctivitis sicca and ichthyosiform dermatosis (Dry eye and curly coat, DE/CC), rod cone dysplasia 4 (PRA-rcd4), spinocerebellar ataxia (SCA); Labrador Retriever (LAB), Cocker spaniel (CKR), Staffordshire Bull Terrier (SBT), Miniature Bull Terrier (MBT), Cavalier King Charles Spaniel (CKCS), Gordon Setter (GDN), Irish Setter (IRISH), Parson Russell Terrier (PRT).
(XLSX)

S4 Table Mutation frequency calculated from registered dogs born per year using all data and testing data only. The estimated mutation frequency derived using data from registered dogs born per year; using test data only (top) and all known genotypes (test and hereditary data—bottom), for each disorder / DNA test per breed; progressive rod-cone degeneration (prcd-PRA), early onset [hereditary] cataract (HC), exercise-induced collapse (EIC), lens luxation (PLL), episodic falling (EF), congenital keratoconjunctivitis sicca and ichthyosiform dermatosis (Dry eye and curly coat, DE/CC), rod cone dysplasia 4 (PRA-rcd4), spinocerebellar ataxia (SCA); Labrador Retriever (LAB), Cocker spaniel (CKR), Staffordshire Bull Terrier (SBT), Miniature Bull Terrier (MBT), Cavalier King Charles Spaniel (CKCS), Gordon Setter (GDN), Irish Setter (IRISH), Parson Russell Terrier (PRT). Grey shading denotes the year the causal mutation was published ('t') as listed in Table 1.
(XLSX)

S5 Table Data from S2-S4 grouped by individual disorder / breed. The results/data described in S2-S4: number of Kennel Club registered dogs born per year, number with known genotype (‘result’—either from a test or hereditary status), number with test results; the number of clear, carrier (‘carr’) and affected (‘aff’) individuals using test data only (‘T_’) and using hereditary, data only (‘H_’); the genotype frequencies and estimated mutation (‘m’) and ‘wild type’ allele (‘wt’) frequencies derived using test data only and all known genotypes (test and hereditary data)–grouped by individual disorder / breed combination; progressive rod-cone degeneration (prcd-PRA), early onset [hereditary] cataract (HC), exercise-induced collapse (EIC), lens luxation (PLL), episodic falling (EF), congenital keratoconjunctivitis sicca and ichthyosiform dermatosis (Dry eye and curly coat, DE/CC), rod cone dysplasia 4 (PRA-rcd4), spinocerebellar ataxia (SCA); Labrador Retriever (LAB), Cocker spaniel (CKR), Staffordshire Bull Terrier (SBT), Miniature Bull Terrier (MBT), Cavalier King Charles Spaniel (CKCS), Gordon Setter (GDN), Irish Setter (IRISH), Parson Russell Terrier (PRT). Grey shading denotes the year the causal mutation was published ('t') as listed in Table 1.
(XLSX)

S1 Fig Plot of decline in mutation frequency on breed population size. Plot and best fit line of decline of POST-TEST_f1 on breed population size (mean registrations, 2000–17). From the regression equation, R-sq was 0.31 and the F statistic was 0.27.
(TIF)

S2 Fig Plot of test uptake on pre-test mutation frequency. Plot and best fit line of test uptake (proportion registered dogs with a result at t+4) on PRE-TEST_f. From the regression equation, R-sq was 0.01 and the F statistic was 0.80.
(TIF)

S3 Fig Plot of decline in mutation frequency on pre-test mutation frequency. Plot and best fit line of decline of POST-TEST_f1 on pre-test mutation frequency (f(m), t-2 to t-4). From the regression equation, R-sq was 0.29 and the F statistic was 0.11.
(TIF)
