# Supplementary material for: Changes in mutation frequency of eight Mendelian inherited disorders in eight pedigree dog populations following introduction of a commercial DNA test
Source: PLoS One. 2019 Jan 16;14(1):e0209864. doi: 10.1371/journal.pone.0209864 (PMC6334900; doi:10.1371/journal.pone.0209864)
Supplement: S1 Table — The numbers of tests per year for each disorder / breed combination; progressive rod-cone degeneration (prcd-PRA), early onset [hereditary] cataract (HC), exercise-induced collapse (EIC), lens luxation (PLL), episodic falling (EF), congenital keratoconjunctivitis sicca and ichthyosiform dermatosis (Dry eye and curly coat, DE/CC), rod cone dysplasia 4 (PRA-rcd4), spinocerebellar ataxia (SCA); Labrador Retriever (LAB), Cocker spaniel (CKR), Staffordshire Bull Terrier (SBT), Miniature Bull Terrier (MBT), Cavalier King Charles Spaniel (CKCS), Gordon Setter (GDN), Irish Setter (IRISH), Parson Russell Terrier (PRT). Grey shading denotes the year the causal mutation was published ('t') as listed in Table 1. (DOCX) [file pone.0209864.s001.docx]

|  | prcd-PRA | | HC | EIC | PLL | EF | DE/CC | PRA-rcd4 | | SCA |
| --- | --- | --- | --- | --- | --- | --- | --- | --- | --- | --- |
| test yr | LAB | CKR | SBT | LAB | MBT | CKCS | CKCS | GDN | IRISH | PRT |
| 2000 | 1 | 0 | 0 | 0 | 0 | 0 | 0 | 0 | 0 | 0 |
| 2001 | 1 | 0 | 0 | 1 | 0 | 0 | 0 | 1 | 0 | 0 |
| 2002 | 0 | 0 | 0 | 0 | 0 | 0 | 0 | 0 | 0 | 0 |
| 2003 | 3 | 2 | 1 | 0 | 0 | 0 | 0 | 0 | 0 | 0 |
| 2004 | 20 | 16 | 0 | 0 | 0 | 0 | 0 | 0 | 0 | 0 |
| 2005 | 32 | 103 | 2 | 0 | 0 | 0 | 0 | 0 | 0 | 0 |
| 2006 | 185 | 156 | 729 | 0 | 0 | 0 | 0 | 0 | 0 | 0 |
| 2007 | 385 | 204 | 423 | 0 | 0 | 0 | 0 | 0 | 0 | 0 |
| 2008 | 485 | 270 | 277 | 10 | 1 | 0 | 0 | 0 | 0 | 0 |
| 2009 | 420 | 367 | 214 | 53 | 104 | 0 | 0 | 0 | 0 | 0 |
| 2010 | 272 | 371 | 180 | 22 | 125 | 0 | 0 | 0 | 0 | 0 |
| 2011 | 311 | 355 | 98 | 53 | 76 | 73 | 71 | 365 | 603 | 0 |
| 2012 | 220 | 247 | 117 | 129 | 52 | 618 | 613 | 83 | 341 | 147 |
| 2013 | 210 | 250 | 101 | 166 | 46 | 500 | 505 | 52 | 184 | 73 |
| 2014 | 170 | 192 | 88 | 227 | 64 | 358 | 356 | 46 | 115 | 27 |
| 2015 | 187 | 146 | 94 | 335 | 30 | 249 | 221 | 30 | 71 | 32 |
| 2016 | 198 | 225 | 72 | 397 | 31 | 238 | 226 | 24 | 62 | 25 |
| 2017 | 164 | 244 | 95 | 451 | 39 | 228 | 209 | 41 | 95 | 22 |
