# Supplementary figures and images for: Changes in mutation frequency of eight Mendelian inherited disorders in eight pedigree dog populations following introduction of a commercial DNA test
Source: PLoS One. 2019 Jan 16;14(1):e0209864. doi: 10.1371/journal.pone.0209864 (PMC6334900; doi:10.1371/journal.pone.0209864)

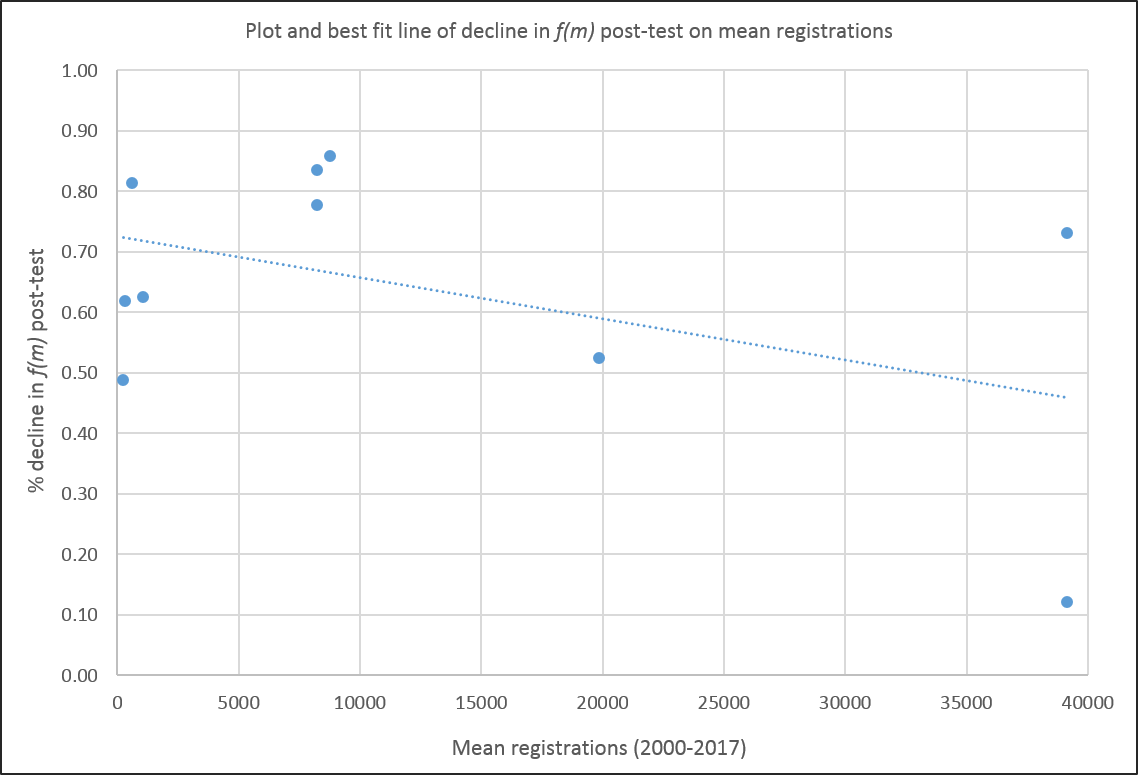

Supplement: S1 Fig — Plot and best fit line of decline of POST-TEST_f1 on breed population size (mean registrations, 2000–17). From the regression equation, R-sq was 0.31 and the F statistic was 0.27. (TIF) [file pone.0209864.s006.tif]

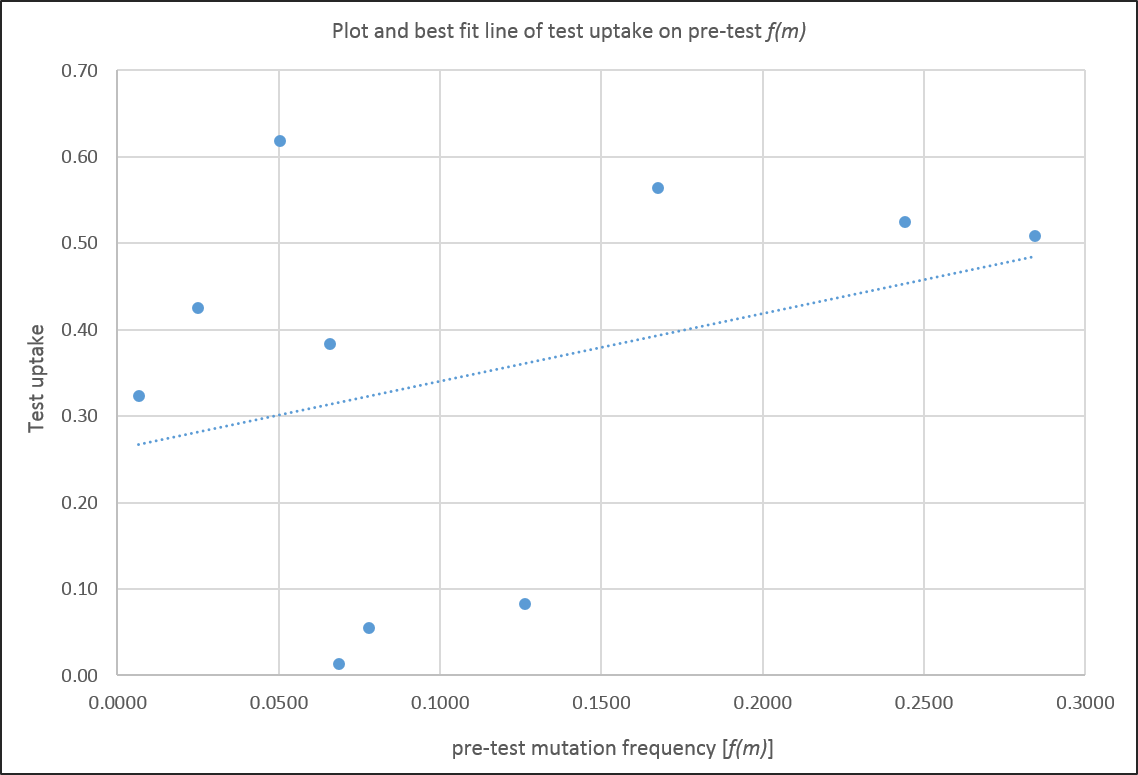

Supplement: S2 Fig — Plot and best fit line of test uptake (proportion registered dogs with a result at t+4) on PRE-TEST_f. From the regression equation, R-sq was 0.01 and the F statistic was 0.80. (TIF) [file pone.0209864.s007.tif]

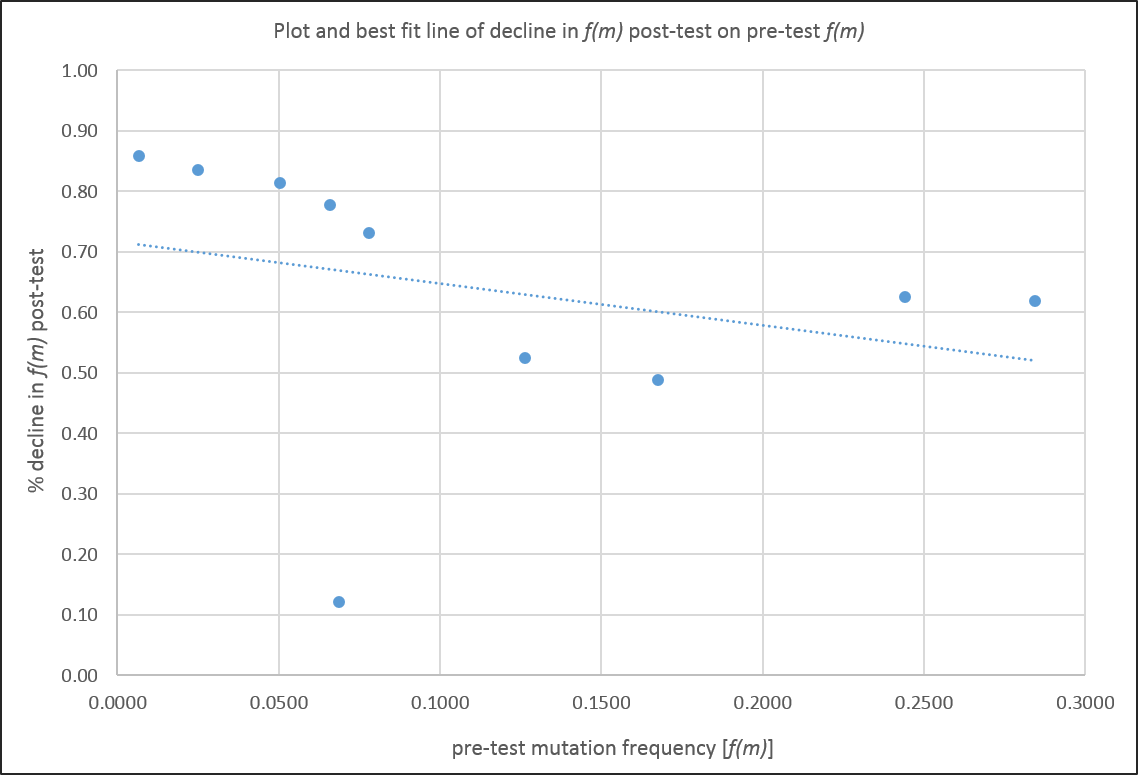

Supplement: S3 Fig — Plot and best fit line of decline of POST-TEST_f1 on pre-test mutation frequency (f(m), t-2 to t-4). From the regression equation, R-sq was 0.29 and the F statistic was 0.11. (TIF) [file pone.0209864.s008.tif]
